# Elevated Risk of HPV‐Associated Oropharyngeal Cancers in Husbands of Women With Anogenital Cancer

**DOI:** 10.1002/cnr2.70333

**Published:** 2025-08-28

**Authors:** Tuomas Lehtinen, Luayo Zhang, Kristina Sundquist, Jan Sundquist, Tim Waterboer, Matti Lehtinen, Kari Hemminki

**Affiliations:** ^1^ FICAN‐Mid Tampere University Tampere Finland; ^2^ Molecular Genetic Epidemiology Division Deutsches Krebsforschungszentrum (DKFZ) Heidelberg Germany; ^3^ Center for Primary Health Care Research Lund University Malmö Sweden; ^4^ Center for Community‐Based Healthcare Research and Education (CoHRE), Department of Functional Pathology, School of Medicine Shimane University Japan; ^5^ Infections and Cancer Epidemiology Division Deutsches Krebsforschungszentrum (DKFZ) Heidelberg Germany; ^6^ Department of Laboratory Medicine Karolinska Institute Stockholm Sweden; ^7^ Faculty of Medicine and Biomedical Center in Pilsen Charles University in Prague Pilsen Czech Republic

**Keywords:** familial cancer, head and neck cancer, viral infection, viral oncology

## Abstract

**Introduction:**

Human papillomavirus (HPV) infection is associated with tonsillar and base‐of‐tongue squamous cell carcinomas (TSCC/BOTSCC). We evaluated the relative risk (RR) of TSCC/BOTSCC in the husbands of women with anogenital cancer using the Swedish family database.

**Methods:**

The Swedish family database includes 3.5 million families and 16 million individuals identified since 1932 and linked to cancer data from 1958 to 2015. We explored the RR of TSCC/BOTSCC in husbands of women diagnosed with anogenital (anal, vulvar/vaginal, cervical) cancer or cervical carcinoma in situ (CIS) as compared to husbands of controls.

**Results:**

The RR of TSCC/BOTSCC in husbands of women with invasive anogenital cancer increased by calendar time and decreasing ages at diagnoses. In 2002–2015, the RR peaked at 5.35 (95% CI 2.21–13.0) in TSCC/BOTSCC cases diagnosed at age < 50 years. In husbands of women with CIS, the RR for TSCC/BOTSCC was 2‐fold irrespective of time period.

**Conclusions:**

Clustering of increased HPV‐associated invasive cancer risk in spouses appears to be calendar‐time dependent. This introduces a new perspective in cancer science: an increase of familial HPV‐associated cancer risk in spouses.

## 1 Introduction

Seventy per cent of oropharyngeal cancers (OPCs), especially tonsillar and base of tongue squamous cell carcinomas (TSCC/BOTSCC) are associated with human papillomavirus (HPV). HPV also causes cervical and other anogenital cancers, respectively 95% (cervical cancer, 30%–85% (vulvar cancer), 65%–90% (vaginal cancer) and 40%–95% (anal cancer)) of which are positive for HPV [1, 2, 3].

In the Nordic countries the incidence of cervical cancer has increased in fertile‐aged women [www.nordcan]. The incidence of OPC has also increased in males under 65 years of age [4]. The husbands of wives with cervical cancer have been reported to have a moderately (two‐fold) increased risk of OPC [5, 6]. We hypothesised that the risk of TSCC/BOTSCC would be especially increased in husbands of women with invasive anogenital cancer or in situ cervical carcinoma (CIS) diagnosed during the last 20 years when they were increasingly likely to pass on infections with oncogenic, high‐risk (hr) HPV types to their partners [6, 7, 8, 9].

## 2 Methods

Family relationships for the Swedish Family‐Cancer Database were obtained from the Multi‐generation Register which contains the Swedish population in families [10]. Cancers were identified from the Swedish Cancer Registry, which was started in 1958 using codes of the International Classification of Diseases version 7 (and later 10). The Swedish family cancer data linked to cancer diagnoses from 1958 to 2015 included some 3.5 million families and 16 million individuals identified since 1932. Its coverage is over 90% of histologically verified incident cancers due to compulsory registration [10, 11]. The 7th or 10th ICD codes were applied in the identification of patients with primary diagnoses of probably HPV‐associated cancers: (1) anogenital: cervix (ICD‐7171, ICD‐10 C53), vulva (1761, C51), vagina (176, C52), anus (1541, C21); 2) base of tongue (141, C01), tongue, other parts (141, C02), tonsil (1450, C09) and oropharynx (145 except 1450, C10).

This register‐based study was approved by the Lund University Ethical Committee (Reg. No. 2012/795), the permission covers involved Swedish research institutes. The Swedish Cancer Registry linkage was conducted in Sweden using pseudonymized codes. The anonymized data which did not allow identification of the cases after the registry linkage was used at the DKFZ, Heidelberg, Germany for the statistical analyses and report writing between 2020–24.

We stratified the couples by decreasing age at the time of diagnosis: (A) husbands (aged > 50 years) of wives (any age) diagnosed with anogenital cancer (A1) or CIS (A2); (B) husbands (aged > 50 years) of wives with anogenital cancer (B1) or CIS (B2) diagnosed < 50 years of age; (C) husbands (aged < 50 years) of wives (any age) diagnosed with anogenital cancer (C1) or CIS (C2) and (D) husbands (aged < 50 years) of wives with anogenital cancer (D1) or CIS (D2) diagnosed < 50 years of age.

Poisson regression modelling was employed to estimate relative risks (RRs) with 95% confidence intervals of OPCs two calendar time strata (1969–01 and 2002–15) by comparing the OPC incidence rates in husbands of women with anogenital cancer or CIS with OPC incidence rates in men whose wives had no anogenital cancer or CIS [12]. All RR estimates were adjusted for age, calendar‐time (5‐year bands), socio‐economic status and residence. *p*‐values for the trends of stepwise RR changes are given.

## 3 Results and Conclusions

**FIGURE 1:**
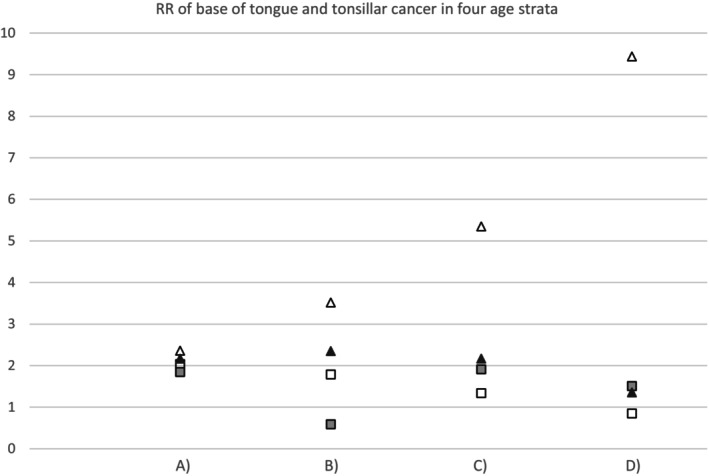
Relative risk (RR) of base of tongue (BOTSCC) and tonsillar cancer (TSCC) in four stepwisely decreasing age strata by calendar‐time: (A) Husbands diagnosed > 50 years of age, (B) husbands diagnosed > 50 years of age, wives diagnosed < 50 years of age, (C) Husbands diagnosed < 50 years of age, (D) Husbands diagnosed < 50 years of age, wives diagnosed < 50 years of age. Symbols: □ = wives diagnosed with invasive anogenital cancer between 1969–2001, Δ = wives diagnosed with invasive anogenital cancer between 2002–2015, ■ = wives diagnosed with in situ cervical cancer between 1969–2001, ▲ = wives diagnosed with in situ cervical cancer between 2002–2015.

In 1969–2001, RR for TSCC/BOTSCC diagnosed in over 50‐year‐old husbands of women with anogenital cancer (Group A1) was 2.0; in 2002–2015 it was 2.4 (Table 1). To evaluate the impact of shorter time from possible hrHPV transmission to the cancer diagnosis, we calculated the RRs for TSCC/BOTSCC diagnosed under 50 years of age (Group C1). These were 1.3 and 5.4 in 1969–2001 and 2002–2015 (Table 1). Furthermore, a statistically significant (*p* < 0.0001) trend of increasing TSCC/BOTSCC RRs by decreasing diagnostic age strata (A1, B1, C1, D1) was observed: 2.36 (95% CI 1.56–3.57), 3.52 (95% CI 1.98–6.26), 5.35 (95% CI 2.21–13.0) and finally 9.44 (95% CI 1.80–49.4) in couples under 50 years of age (Figure 1).

**TABLE 1:** Calendar time‐dependent relative risk (RR) and 95% confidence interval (95% CI) for oropharyngeal cancer* ^,^ ^b^ (OPC) diagnosed in (A) husbands aged > 50 years of wives diagnosed with (1) invasive anogenital cancer, (2) cervical in situ carcinoma (CIS); (B) husbands aged > 50 years of wives diagnosed < 50 years with (1) invasive ano anogenital cancer, (2) CIS; (C) husbands aged < 50 years of wives diagnosed with (1) invasive anogenital cancer, (2) CIS; (D) husbands aged < 50 years of wives diagnosed < 50 years with (1) invasive anogenital cancer, (2) CIS.

OPC	1969–2001			2002–2015		
	Cases	RR	95% CI	Cases	RR	95% CI
(A1) Husbands (aged > 50 years) of wives diagnosed with invasive anogenital cancer						
TSCC/BOTSCC^a^	6	2.04	0.91–4.57	23	2.36	1.56–3.57
Oropharynx&tongue other parts^b^	5	0.61	0.25–1.47	22	1.71	1.12–2.60
Overall	11	0.99	0.55–1.79	45	1.99	1.48–2.67
(A2) Husbands (aged > 50 years) of wives diagnosed with in situ cervical cancer						
TSCC/BOTSCC^a^	35	1.85	1.33–2.70	204	2.17	1.87–2.51
Oropharynx&tongue other parts^b^	77	1.69	1.34–2.17	168	1.56	1.32–1.83
Overall	112	1.73	1.44–2.15	372	1.85	1.65–2.05
(B1) Husbands (aged > 50 years) of wives diagnosed with invasive anogenital cancer < 50 years of age						
TSCC/BOTSCC^a^	4	1.79	0.61–5.27	16	3.52	1.98–6.26
Oropharynx&tongue other parts^b^	1	0.37	0.05–2.77	13	2.04	1.12–3.70
Overall	5	1.01	0.40–2.55	29	2.66	1.77–4.02
(B2) Husbands (aged > 50 years) of wives diagnosed with in situ cervical cancer < 50 years of age						
TSCC/BOTSCC^a^	32	0.59	0.40–0.83	194	2.35	1.24–4.44
Oropharynx&tongue other parts^b^	67	1.33	1.02–1.70	153	1.62	1.37–1.91
Overall	99	0.92	0.75–1.13	347	1.93	1.25–3.02
(C1) Husbands (aged < 50 years) of wives diagnosed with invasive anogenital cancer						
TSCC/BOTSCC^a^	2	1.34	0.33–5.40	5	5.35	2.21–12.97
Oropharynx&tongue other parts^b^	2	0.48	0.12–1.92	1	1.07	0.15–7.65
Overall	4	0.72	0.27–1.91	6	3.21	1.43–7.18
(C2) Husbands (aged < 50 years) of wives diagnosed with in situ cervical cancer						
TSCC/BOTSCC^a^	26	1.91	1.25–2.88	31	2.17	1.48–3.20
Oropharynx&tongue other parts^b^	45	1.08	0.79–1.47	14	0.84	0.47–1.50
Overall	71	1.28	1.00–1.64	45	1.49	1.08–2.04
(D1) husbands (aged < 50 years) of wives diagnosed with invasive anogenital cancer < 50 years of age						
TSCC/BOTSCC^a^	1	0.85	0.11–6.75	3	9.44	1.80–49.38
Oropharynx&tongue other parts^b^	1	0.31	0.04–2.33	1	1.94	0.21–18.31
Overall	2	0.44	0.10–1.84	4	5.06	1.42–18.02
(D2) Husbands (aged < 50 years) of wives diagnosed with in situ cervical cancer < 50 years of age						
TSCC/BOTSCC^a^	25	1.51	0.98–2.23	31	1.36	0.92–1.94
Oropharynx&tongue other parts^b^	40	2.21	1.58–3.03	14	0.59	0.30–1.04
Overall	65	1.84	1.39–2.33	45	0.99	0.71–1.34

^*^ 95% Confidence intervals with lower limit above 1.0 indicate statistically significant RR estimates following a common practice.

^a^ TSCC (tonsil, 1450, C09) and BOTSCC (base of tongue, C01).

^b^ Tongue, other parts (C02) and oropharynx (C10).

Among husbands of women with CIS, corresponding moderate RR estimates varied between 1.9 and 2.2 (Table 1) without trends. The prevalence of oral HPV infections is higher in women with cervical HPV infection than without it [13]. Moreover, the higher the HPV DNA load in the cervix is, the stronger the association between HPV and cervical carcinoma in situ [14]. Thus, husbands of women with CIS probably got an infection with a hrHPV type from their spouses. However, half of the CIS cases do not progress into invasive cancer, perhaps due to a larger proportion of less oncogenic hrHPV types (other than HPV16 being) involved in CIS than in invasive cancer [1, 2, 3, 14, 15, 16]. Possible intertypic differences in the oncogenicity of hrHPV subtypes transmitted by women with CIS as compared to those transmitted by women with invasive cancer may further explain the difference in the familial risk estimates between the two different types of couples: comprising wives with CIS vs. wives with invasive anogenital cancer.

Most notably, the RRs increased from 1969–2001 to 2002–2015, and peaked at 5.35 (*p* < 0.05, Table 1) in the youngest TSCC/BOTSCC cases (< 50 years of age) with wives diagnosed with invasive anogenital cancer between 2002–2015. This result, even if based on a limited number of observations, is in line with our a priori hypothesis that increased exposure to hrHPV infections following the sexual revolution has resulted in an increased risk of HPV‐associated cancers in both genders.

The mostly HPV‐associated OPCs, i.e., tonsillar and base of tongue cancers, are anatomically differentiated from non‐HPV‐associated OPCs [1]. Furthermore, the clinical features of the HPV‐associated OPCs also differ from non‐HPV‐associated OPCs [17]. These facts further emphasise the importance of studying these two HPV‐associated OPCs (TSCC/BOTSCC) separately in this familial setting. The Swedish high‐quality registry data (sizeable cohort with ample power for over time analysis) provided a unique opportunity for this.

As our study is anonymous and registry‐based, it has its limitations. Most notably, the HPV infection status and genotype (even if most probably HPV16) of the cases remain unknown. The population‐based Swedish family database [10] linked to cancer diagnoses does not have data on risk‐taking sexual behaviour, which could not be adjusted for. Relying on the ICD codes can be limited from the anatomical perspective, but ≥ 70% of TSCC/BOTSCC cases identified during the last 10 years have been HPV positive [18]. Small numbers in the invasive cancer group is a limitation.

The identified increase in the familial RR estimates over calendar time fits the continuous increase in the incidence and prevalence of HPV in TSCC and BOTSCC in Sweden [17]. Moreover, both the hrHPV prevalence in the fertile‐aged populations and the incidence of cervical cancer have been increasing in the Nordic countries up to the establishment of HPV vaccination programs [8, 9, 19], www.nordcan.se. Compiling data on the peak incidence calendar time period and the youngest cases of the most HPV‐associated male TSCC/BOTSCC cases and female anogenital cancers together, we are now introducing a new perspective in cancer science: increase of familial HPV‐associated cancer risk in spouses.

Finally, because of the long incubation period from persistent HPV infection to OPC and changes in sexual practices, especially the increase in the practice of oral sex, middle‐aged individuals probably continue to have an increased risk of familial HPV‐associated cancers in the foreseeable future. Studies on the implementation of HPV‐based OPC screening are warranted to prevent imminent epidemics of HPV‐associated OPCs in the middle‐aged population [20].

## Author Contributions

**Tuomas Lehtinen:** conceptualization (equal), visualization (lead), writing – original draft (lead). **Luayo Zhang:** data curation (lead), formal analysis (lead), writing – review and editing (equal). **Kristina Sundquist:** resources (equal), writing – review and editing (equal). **Jan Sundquist:** resources (equal), writing – review and editing (equal). **Tim Waterboer:** conceptualization (equal), writing – review and editing (equal). **Matti Lehtinen:** conceptualization (equal), writing – review and editing (equal). **Kari Hemminki:** supervision (equal), writing – review and editing (equal).

## Conflicts of Interest

The authors declare no conflicts of interest.

## Data Availability

Data available on request due to privacy/ethical restrictions.
